# Comparing the effectiveness of robot-based to human-based intervention in improving joint attention in autistic children

**DOI:** 10.3389/fpsyt.2023.1114907

**Published:** 2023-05-05

**Authors:** Wing-Chee So, Wing-Wun Law, Chun-Ho Cheng, Cassandra Lee, Ka-Ching Ng, Fai-Yeung Kwok, Ho-Wai Lam, Ka-Yee Lam

**Affiliations:** Department of Educational Psychology, The Chinese University of Hong Kong, Hong Kong, Hong Kong SAR, China

**Keywords:** social robots, autism, joint attention, intervention, childhood

## Abstract

**Background:**

Children with autism have impairments in initiation of joint attention (IJA) and response to joint attention (RJA).

**Aims:**

The present study compared the learning effectiveness of robot-based intervention (RBI) with that of content-matched human-based intervention (HBI) in improving joint attention (JA). We examined whether RBI would enhance RJA, in comparison to HBI. We also examined whether RBI would increase IJA, in comparison to HBI.

**Methods and procedures:**

Thirty-eight Chinese-speaking children with autism aged 6 to 9 years were randomly assigned to RBI and HBI groups. Before intervention, their autism severity, cognitive abilities, and language skills were assessed. Each child received six 30-min training sessions over 3 weeks. During training, he/she watched one or two robot/human dramas twice where two robot/human actors demonstrated eye contact and RJA.

**Outcomes and results:**

Children in the RBI (but not HBI) group produced more RJA and IJA behaviors in the delayed post-test than in the pre-test. Parents of the RBI children rated the program more positively than those of the HBI children.

**Conclusions and implications:**

RBI may be more effective than HBI in promoting JA in autistic children with high support needs. Our findings shed light on the application of robot dramas in enhancing social communication skills.

## Introduction

Autism spectrum disorder (ASD) is a complex and pervasive neurodevelopmental disorder. The prevalence of children with ASD is increasing in Hong Kong and around the world. In 2021, the Centers for Disease Control and Prevention (CDC) reported that approximately 1 in 44 children in the U.S. is diagnosed with ASD, based on 2018 data. According to the Education Bureau in Hong Kong, the number of primary and secondary school students diagnosed with autism increased 60% from 2015 to 2022, reaching a total of 12,367 students. The male to female ratio of children with autism is 5:1, with 1 in 42 boys diagnosed with autism, compared to 1 in 189 girls. No cure exists for ASD. Treatment success lies in behavioral support in social communication skill development and reduction in restricted interests and repetitive and challenging behavior.

Among various approaches, a notable treatment for people with ASD is that of applied behavior analysis (ABA). ABA has become widely accepted among healthcare professionals and is used in many schools and treatment clinics. However, ABA relies heavily on human therapists and parents, as they design and implement suitable interventions and evaluate the learning outcomes. According to the Social Motivation Theory of Autism, individuals with ASD show deficits in orienting themselves toward social stimuli, engaging with humans, and maintaining social relations (1). Therefore, these individuals may not be responsive to their human therapists. Furthermore, according to Markram and Markram’s (2) Intense World Theory, individuals with ASD tend to have excessive reactivity and rapidly form memories of experiences due to a particular form of brain hypertrophy. The dynamic facial features and expressions of human beings may induce intensive sensory processing in individuals with ASD, possibly resulting in sensory and emotional overstimulation and distraction. As a result, individuals with ASD may actively avoid sensory stimulation, instead focusing on elementary features that are more predictable, which may interfere with their learning. Additionally, human-based intervention incurs high costs to families with children with ASD. In Hong Kong, the cost of one-to-one treatment by behavioral therapists is about USD100 an hour (3). Parents of ASD children, therefore, face extreme financial pressure. As a result, it is necessary to develop an effective, affordable, and timely intervention for children with ASD.

The potential limitations of human-based intervention have given rise to the application of technology, such as social robots, in autism therapies. Social robots are robots designed to interact with humans and other robots in a socially acceptable fashion, express emotions and intention in a human-perceptible way, and resolve goals with fellow human or robot agents (4, 5). The use of social robots in autism therapy is theory driven. Based on the empathizing-systemizing theory (6), robots are operated on predictable and lawful systems, thereby providing children with ASD with a highly structured learning environment and helping them to focus on the relevant stimuli. Additionally, children with ASD do not need to consider socio-emotional expectations when interacting with robots (7), thus reducing their social anxiety (8). Social robots can also be massively manufactured at a fixed cost. Thus, they can provide timely and affordable interventions to the children with ASD.

In spite of the potential strengths of robot-based intervention in autism therapy, its effectiveness, in comparison to that of human-based intervention, in improving social and communication skills in children with autism, has been understudied. This study aimed to fill this gap in the literature by developing a robot-based intervention for improving joint attention in children with ASD and comparing its learning effectiveness with that of human-based intervention. Joint attention is the ability to share attention with others through pointing, showing, and coordinating looks between objects and people (9–12). There are two classes of joint attention: (1) response to joint attention (RJA), which is defined as a child’s response in following another person’s shift in eye gaze and/or pointing; and (2) initiation to joint attention (IJA), which is defined as a child’s ability to spontaneously seek another’s attention or direct the attention of others to share their experience of an object or event through eye gaze and/or pointing.

Children with ASD have RJA and IJA impairments in the first 2 years of life (9–12). These impairments are still evident during the preschool period (e.g., 13, 14), leading to delays in social cognitive (15, 16) and language development (10, 18). Children with autism, particularly those with high support needs, have more severe impairments in IJA and RJA than their counterparts with low support needs (19, 20).

In a meta-analysis conducted by Murza et al. (21), nine studies adopting a joint attention treatment versus control design were aggregated for treatment effect, with Hedges g = 0.660, 95% CI [0.395, 0.925], *p* < 0.001. These findings suggested there was an improvement in joint attention of approximately two-third’s SD in treated children in comparison with those in the control condition. Of these studies, several of them explicitly and directly taught children with ASD how to initiate or respond to joint attention bids, yielding promising results (e.g., 22–24).

The aforementioned studies relied heavily on human therapists, who were greatly involved in the intervention procedures and provision of feedback and reinforcement. Recently, six studies conducted by different research groups examined whether social robots could improve joint attention but reported mix findings. Kumazaki et al. (25) deployed a social robot, CommU (a robot with big eyes and a head that are movable), for ASD and typically developing children with normal cognitive functioning. In the robotic intervention group, the participants interacted with a human agent, followed by CommU, and finally the human agent again, for 5 minutes each. During the “CommU” session, the child was sitting in front of the robot, which was placed on a table in the middle of the room. In the control group, the participants interacted only with the human agent throughout the experiment. During the interaction, CommU or the human agent greeted the child and asked him/her questions (e.g., “Do you like juice?”), and then gazed to the left or right for 3 seconds. RJA was coded when the child responded to (i.e., turned to look at) the correct target within the 3 s window. The findings showed that children with ASD demonstrated better RJA under the CommU condition than under the human agent condition. More importantly, through practicing RJA with CommU, children with ASD exhibited improvement in RJA tasks with humans.

Different from Kumazaki et al. (25), So et al. (26) adopted a relatively indirect approach to teach young autistic pre-schoolers with low support needs IJA and RJA. They programmed two NAO robots to speak and/or produce pretend acts in three different dramas. After watching each drama twice, the child was then invited to act as one of the characters and participate in a role-play with the robot. Then he/she swapped roles and acted as the other character with the robot. Finally, the child was invited to take part in role-plays with a human experimenter for both characters. In the play-drama intervention protocol, children were expected to discover the implications of their play behaviors and the impacts of their responses on others, and hence be motivated to initiate joint attention with others and to understand how to respond to others’ joint attention behaviors. Children in the intervention group took robot drama classes, while those in the waitlist group received the intervention after the completion of the research. The results showed that, while children in both groups had already reached a high level of RJA in the pre-test, the children in the intervention condition produced significantly more IJA in the post-test than in the pre-test. Both RJA and IJA were measured by Early Social Communication Scale (ESCS; 27).

In another study, So et al. (28) also adopted a robot-drama approach, but time programmed a different social robot, HUMANE. Different from the previous study, in which joint attention (JA) was not directly and explicitly taught, the HUMANE robots in this study demonstrated RJA explicitly in the dramas. Eighteen Chinese-speaking children aged 6 to 8 with autism and moderate intellectual disabilities participated and received robot drama intervention at staggered time points. Their RJA, measured by ESCS, improved after taking six robot drama lessons. Even more promising, their positive learning outcomes were maintained for 1 to 2 months. In addition to RJA, the IJA ability of all children was enhanced and maintained over time despite IJA not being explicitly taught. While So et al. (26, 28) reported effective learning outcomes of robotic intervention in their previous studies, they did not include the human intervention condition for comparison.

Alternatively, two other studies found insignificant effects of robotic intervention in improving JA. In one of the studies, Zheng et al. (29) deployed an NAO robot and recruited 23 children aged 1 to 3 years old with ASD and IQs around 60. Children in the intervention condition sat in front of the NAO, which directed their attention to two computer monitors hung in the treatment room by looking at and/or pointing to the target monitor and saying, “Look over there!.” The attention-tracking sub-system detected the children’s attention to the robot and the targets using a camera array-based algorithm and rewarded the children (e.g., showed video clips) if they responded accurately to an attention prompt. Their findings showed a non-significant difference in RJA between the children who received the intervention and those who did not. However, the insignificant finding might be attributable to two reasons. First, there were large individual variations in the children’s responses to the NAO during the intervention. Second, children in general might simply not understand why the NAO suddenly looked at one of the monitors. Understanding the intention of the NAO’s IJA might have facilitated the children’s RJA. For example, the children participating in Kumazaki et al.’s (25) study did follow the eye gaze of CommU because CommU first conversed with the children before directing them to look in a particular direction.

In another study, Srinivasan et al. (30) and Srinivasan et al. (31) examined the effects of rhythm and robotic interventions on the JA, measured by Standardized test of Joint Attention (JTAT) (32), of 36 school-aged children with ASD and development delays. These children were randomly assigned to three conditions: rhythm intervention, robot intervention, and care-as-usual (e.g., ABA treatment). The rhythm and robot groups engaged in whole-body synchrony and imitation-based games, with the rhythm therapy delivered primarily by a human and the robot therapy delivered mainly by an NAO (controlled by a human). Their results showed that there were significant improvements in JA between the pre-test and the post-test in the rhythm and care-as-usual groups but not in the robot intervention group. However, the results of these three groups could not be compared because the whole-body movements involved in the rhythm and robot interventions were different, due to the technological constraints of the NAO.

One recent study has applied robot-assisted training to the clinical setting and found that the combination of robot-assisted training with standard therapy was more effective than the standard therapy alone in making behavioral requests and initiating social interactions but not in initiating and responding to joint attention (33). In their study, autistic children aged 4 to 7 with different severity levels of autism were randomly assigned to two intervention conditions, combined therapy, and standard therapy alone. In the combined therapy condition, healthcare professionals delivered both robot-mediated activity and standard therapy. In each of the robot-mediated sessions, a small commercial toy robot, Cozmo (Anki Robotics, San Francisco, CA, USA), turned towards one of the two cubes and looked at it for a few seconds before returning to the original position. Then Cozmo looked back to the child, who was then asked by the healthcare professional which cube Cozmo looked at. Using ESCS, their findings have shown that autistic children got improvements mainly in initiating behavioral requests and interacting with others through pointing and eye gaze.

To sum up, researchers have not yet reached a consensus on the effectiveness of robots in improving JA in children with ASD. While So et al. (26, 28) found positive learning outcomes of robot dramas, they did not include human-based intervention for comparison of learning effectiveness. Additionally, very few studies examined whether robotic intervention is more effective than human intervention in promoting JA (25, 30, 31). The findings of these studies, however, should be interpreted with caution because the designs of their robotic intervention might not be comparable to human-based intervention.

Given the limitations of the research designs of the previous studies, the present study programmed two HUMANE social robots to perform dramas modeling RJA for school-aged autistic children with high support needs (Iqs < 69) (28), as well as designing a comparable human-based intervention, in which two human actors performed the same dramas to another group of age-and IQ-matched children with ASD and followed the same procedures as the robot-based intervention. Keeping the contents and procedures of both robot and human dramas the same is crucial for comparing the effectiveness of these two kinds of interventions in improving JA. IJA and RJA of both groups of children would be assessed and compared before and after the interventions. Any differences in the learning outcomes in IJA and RJA would then be attributable to the teaching agent (robots vs. humans). We examined whether children receiving the robot-based intervention would have their RJA improved, in comparison to those in the human-based intervention. We also examined whether our robot dramas, which were originally designed for RJA, would yield a positive impact on IJA in the children in the robot-based intervention group.

In the past decades, researchers and clinicians have raised concerns on the ethical and legal issues related to the robot-based intervention and the data collection associated with this (e.g., 34–36). One of the ethical issues concerns privacy. Robots that have their sensors enabled can detect and store children’s behavioral data during the intervention. As a result, public would concern where these data should be stored and who should be granted access of the data. In the present study, the sensors of the robots were disabled, and the robot dramas were pre-programed. Therefore, there seems little reason to worry about the privacy. Another ethical concern is the control and power of the robots that may restrict humans’ activities and impact their cognition and decision making. The robots deployed in this study were fully programmed and operated by the human therapists, instead of vice versa. In other words, the human therapists had 100% control over the robots. To this end, ethical concerns associated with the robot-based intervention seem to be minimal in the present study.

## Method

### Research method

A quasi-experimental pretest-posttest repeated measures design was adopted. We recruited children participants who were readily available in a special school that mainly educates children with IQs below 70 (convenient sampling). We then randomly assigned them to one of the two interventions: human-based intervention or robot-based intervention. Children in both groups had their IJA and RJA evaluated before (pre-test) and after (post-test) after the intervention using ESCS. Parents’ ratings on the intervention programs were collected after the intervention. Repeated measures ANOVA analyses were conducted.

### Participants

A total of 38 Chinese-speaking (Cantonese-speaking) children aged between 6 and 9 years old (7 females) participated in this study. All children participating in the study had been diagnosed with autism between the ages of 18 and 36 months by pediatricians at the Child Assessment Centre for the Department of Health in Hong Kong, who used the fourth edition of the Diagnostic and Statistical Manual of Mental Disorders (DSM-IV-TR; 37). Their ASD diagnoses were further confirmed by the research team using the Autism Diagnostic Observation Schedule – Second Edition (ADOS-2; 38). Thus, all children met the ADOS-2 and DSM-IV-TR criteria for ASD. Their diagnoses remained unvaried using the DSM-5 (39). They all studied in the same special needs school enrolled in by students with moderate intellectual disabilities during the time of the present study. Thus, besides autism, these students also have intellectual disabilities. They were randomly assigned to two conditions: robot-based intervention and human-based intervention. The mean age of children in the robot-based intervention group was 7.51 (*N* = 19; SD = 0.87; 4 females) and that of children in the human-based intervention group was 7.91 (*N* = 19; SD = 0.89; 3 females), *t* (37) = 1.43, *p* < 0.16. Children assigned to the robot-based intervention watched dramas acted out by robots while their peers in the human-based intervention watched human dramas.

### Pre-intervention assessment

At the beginning of the experiment, all the children and their parents took various assessments in order to examine whether there were individual differences in autism severity, cognitive abilities, and language skills, which might influence the learning outcomes. The children took ADOS-2 (Module 2), the Childhood Autism Rating Scale™ -- Second Edition (CARS™-2; 40), and the Kaufman Brief Intelligence Test—Second Edition (KBIT-2; 41). ADOS-2 assesses and diagnoses autism spectrum disorders across age, developmental level, and language skills (38). It was conducted by a trained professional who had completed ADOS-2 Advanced/Research Training. The children’s comparison scores were reported. CARS™-2 measures the severity level of autism. The children’s raw scores were reported. KBIT-2 assesses both verbal and nonverbal intelligence in people from 4 through 90 years of age. It is composed of two separate scales. The Verbal Scale contains two kinds of items—Verbal Knowledge and Riddles—both of which assess crystallized ability (knowledge of words and their meanings). The Nonverbal Scale includes a Matrices subtest that assesses fluid thinking—the ability to solve new problems by perceiving relationships and completing analogies. The test items are free of cultural and gender bias. The children’s composite IQ was reported. Caregivers of the participating children were also invited to complete two questionnaires: the Social Communication Questionnaire (SCQ; 42) and the school-age form of the Social Responsiveness Scale – Second Edition (SRS-2^nd^ Edition; 43). SCQ includes 40 questions and helps evaluate communication skills and social functioning in children. The school-age form of SRS-2^nd^ Edition has 65 questions scored 0 to 3 on a Likert-type scale, which identifies the severity of social impairments in individuals with ASD. The raw scores from SCQ and SRS-2^nd^ Edition were reported.^1^

Table 1 shows both groups of children’s chronological ages and descriptive statistics regarding their performances in each assessment. Children of both groups were confirmed as having autism (average ADOS-2 comparison score was 8; average CARS™-2 was approximately 30). All children had their ADOS-2 score higher than the cut-off (i.e., 7). Additionally, they had severe social impairments (average raw score of SRS-2^nd^ Edition >100). They might also have had moderate intellectual disabilities (average composite IQ score of KBIT-2 around 40).

**Table 1:** Descriptive statistics of assessment performance of children with ASD in both conditions.

Condition	Chronological age	SCQ	SRS-2^nd^ Edition	CARS™-2	ADOS-2	KBIT-2
Robot-based	7.51 (SD = 0.87)	20.76 (SD = 6.13)	106.06 (SD = 30.02)	29.03 (SD = 3.66)	8.28 (SD = 2.44)	44.33 (SD = 9.81)
Human-based	7.91 (SD = 0.89)	22.20 (SD = 4.29)	101.05 (SD = 23.07)	30.27 (SD = 3.43)	8.05 (SD = 2.36)	40.83 (SD = 2.43)
*t*	1.43	0.84	0.57	1.06	0.30	1.47
*p* value	0.16	0.41	0.57	0.30	0.77	0.15

SCQ: Social Communication Questionnaire (42); SRS-2^nd^ Edition: Social Responsiveness Scale – Second Edition (43); CARS™-2: Childhood Autism Rating Scale™ – Second Edition (40); ADOS-2: Autism Diagnostic Observation Schedule – Second Edition (38); KBIT-2 [Kaufman Brief Intelligence Test – Second Edition; (41)].

### Stimuli

#### Prescribed dramas

We adopted the six dramas used in So et al.’s (28) study. All six dramas covered different scenarios to facilitate generalization of acquired skills to different contexts (e.g., toy store, home). Each drama lasted for approximately a minute and was shown twice. Pictures of objects (e.g., red train, slide) were also presented during the intervention.

In each of the dramas, Dash and Skye were the only two characters. Skye behaved like an individual with autism but, luckily, he had a good friend, Dash, who taught him appropriate social skills. In one of the dramas modeling RJA, Dash and Skye were in a park, and they discussed what to play. Dash was looking at the swing and said it should be fun. However, Skye did not follow the eye gaze of the Dash and he looked at the slide, saying, “How about we play the slide?” Then Dash corrected him and said, “Look at me! I am looking at the swing.” Skye then looked at Dash and turned his eyes to the swing and conversed with Dash again. The contents of dramas were the same in the human-based and robot-based interventions except that the characters were portrayed by two human researchers in the human-based intervention and by robots in the robot-based intervention. Both robots were controlled by a human researcher through separate laptops.

#### Social robots

For the robot-based intervention, we programmed two HUMANE social robots to act in the dramas and model RJA behaviors. HUMANE has been used in autism therapy (44). It is approximately 25 cm tall and weighs 3.2 kg. It was deployed in the present study because its head movements and eye gaze can be programmed to produce various IJA and RJA behaviors (e.g., shifting eye gaze from one object to another).

### Procedures

The intervention and pre-and post-test assessments were conducted in the treatment room at a special school for students aged 6 to 18 with moderate intellectual disabilities. The treatment room was often used by the children for school activities. The room was equipped with two HUMANE robots and two cameras in front of the child in the robot-based intervention, and with two cameras only in the human-based intervention. The cameras recorded the speech and hand movements the child produced during the sessions.

#### Pre-test

We used the Early Social-Communication Scales (ESCS; 27). ESCS has shown good reliability and validity in a variety of studies (17, 20). While ESCS is designed to evaluate social skills in children aged 8 to 30 months, it is commonly administered for children with neurodevelopmental disorders even though their chronological age is beyond 30 months. ESCS is composed of three components: Social Interaction, Joint Attention, and Behavioral Requests. The present study focused on the dimension of joint attention. This subscale was administered before the intervention. One experimenter, who was trained to administer ESCS and naïve to the research hypothesis, conducted this task. She followed the procedures established in the manual (27). During assessment, the experimenter and the child sat facing each other at a table with a set of toys in view but beyond the reach of the child. The toys included several small wind-up and hand-operated mechanical toys, a hat, a comb, glasses, a ball, a car, a balloon, a book, and four posters. The child was, one at a time, presented with three trials of the wind-up mechanical toys, three trials of the hand-operated toys, two trials of a social interaction game, eight trials of pointing to the posters, and six trials of pointing to the book. Each trial, the experimenter referred the toys with an open hand gesture to the toys and asked, “What do you want to play with?” Then the experimenter waited for 3 seconds (silent count instead of using a stopwatch/clock). If the child initiated a joint attention bid (e.g., coordinated looking, pointing, and showing), the experimenter presented him/her the chosen toy and said, “Here it is.” The experimenter should not label the toy, request that the child do something with it, or use the words like “see” or “look.” If the child did not initiate joint attention at all, the experimenter presented one for him/her. This was the time when the child might respond to the experimenter’s pointing and gaze (i.e., response to joint attention). The session lasted for 15 to 20 min and was videotaped. Each child’s IJA (coordinated looking, pointing, and showing) and RJA (responding to the experimenter’s pointing and gazing) were then coded by one coder who were unaware of the hypotheses of the present study. All IJA and RJA behaviors were then summed up. We did not separate the IJA and RJA behaviors for different toys because it was not expected that the types of toys would influence the JA behaviors.

Fidelity checks were conducted. A group of two expert researchers, who had more than 2 years of administering ESCS, viewed all the ESCS videos and independently evaluated whether the trained experimenter adhered to the designated procedures when administering the scale to each child (45). On average, the trained experimenter followed the procedures 92.53% of the time across all children. Since the overall fidelity scores were above 85%, it was valid to use all ESCS data for further coding and analysis.

We established the reliability of our measures by asking a second coder to transcribe 20% of the videotaped sessions. Agreement between the coders was 92.89% for the identification of IJA (Cohen’s Kappa = 0.91, *p* < 0.001), and 95.37% for the identification of RJA (Cohen’s Kappa = 0.94, *p* < 0.001).

#### Training

After the pre-test, children in both conditions received training. Each child received six training sessions, with each session lasting for 30 min, twice per week, over 3 weeks. The training sessions were held every two to 3 days within a week. He/she watched one or two robot/human dramas twice in each training session depending on his/her attention span. In total each child watched six dramas in this intervention. Each session was videotaped for fidelity checks and coding of behaviors. Two cameras were placed at both corners of the room. In each robot drama training session, the human researcher sat next to the child and controlled the social robots, Dash and Skye, remotely by using a laptop. Both robots were placed on the table, which was approximately 1 meter away from the child. The human researcher was sitting next to the robots.

**Figure 1:**
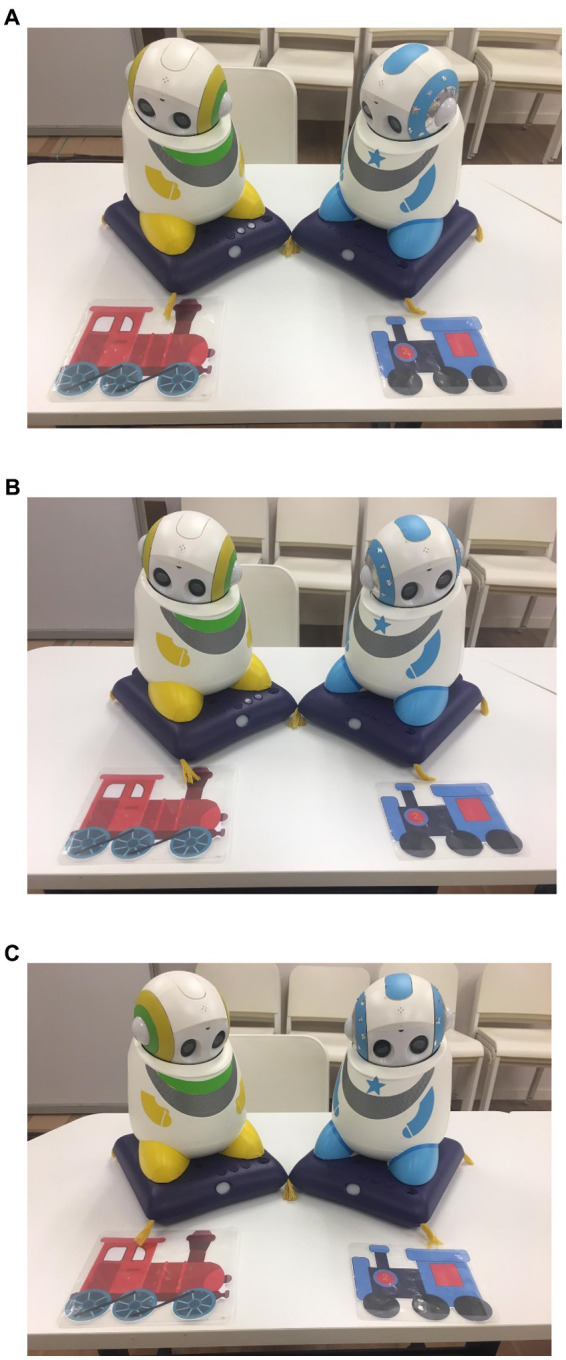
Robot drama demonstrating the two robots looking at the red train **(A)**, followed by Dash switching his eye gaze to the blue train **(B)**, and the two robots looking at the blue train **(C)**. In each of the dramas, one robot acted as Dash and another as Skye. In one of the dramas modeling RJA, Dash and Skye initially were talking about the red train (Starting scene: **A**). Both looked at the red train and said, “This train is awesome!” Then Dash switched his eye gaze to the blue train and said, “I also like this one!” (Middle scene: **B**). As Skye did not follow the eye gaze of Dash, he thought Dash was still talking about the red train. Dash corrected him and said, “Look at me! I am looking at the blue train.” Skye then looked at Dash and turned his eyes to the blue train and conversed with Dash again (Last scene: **C**). This figure is derived from an article published in Disabilities and Rehabilitation: Assistive Technology, Feb 2023, copyright Taylor and Francis, available online: https://www.tandfonline.com/doi/full/10.1080/17483107.2020.1841836.

One of the robots greeted the child, then gave the following instructions: “Today, we are going to perform a drama. Please sit back, relax, and watch our drama.” Then Dash and Skye started acting and conversed with each other until Skye lost track of Dash’s eye gaze and misinterpreted what Dash was talking about or what he was interested in (see Figures 1A–C). Next, the human researcher asked the child to point to the objects Skye and Dash were looking at in order to ensure the child was aware that Skye and Dash were looking at different objects (e.g., Skye was looking at the red train while Dash was looking at the blue train). The child was prompted by the human researcher when he or she did not respond in 3s. If the child’s responses were accurate, he/she proceeded to watch the rest of the drama where Dash corrected Skye and Skye learnt to follow Dash’s eye gaze. If his/her responses were inaccurate, the human researcher would provide the correct answers. At the end of the drama, the human researcher also asked the child to point to the object Skye and Dash were both looking at (e.g., blue train). The same procedures were adopted for other dramas. Each drama was performed twice.

**Figure 2:**
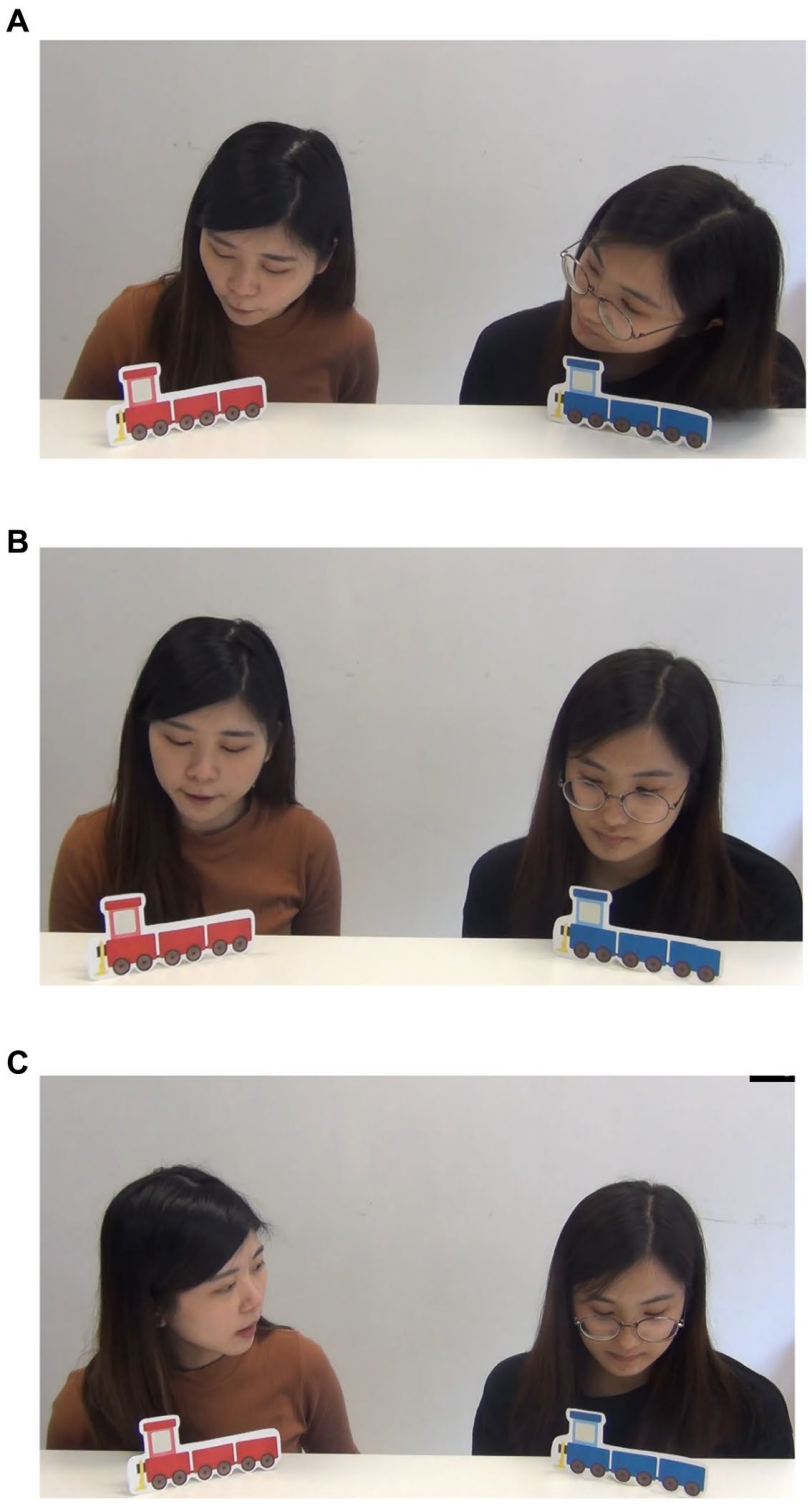
Human drama demonstrating the two humans looking at the red train (Starting scene: **A**), followed by one human researcher switching her eye gaze to the blue train (Middle scene: **B**), and the two humans looking at the blue train (Last scene: **C**). The contents of the human dramas were the same except that the characters (Mary and Ann) were portrayed by two human researchers (see figures **A–C**).

The procedures of the human drama lessons were the same as those of the robot drama lessons (Figures 2A–C). One human researcher sat next to the child while the other two acted as Mary and Ann. The two human actors were trained before the intervention. One of the human actors greeted the child and then began the drama. The human researcher then asked the child questions and prompted him/her if necessary. Fidelity checks were conducted for both the robot and the human dramas. A group of two expert researchers, who had more than 2 years of administering related intervention, viewed all the videos of intervention and independently evaluated whether the robots or human researchers adhered to the designated procedures (45). On average, 96.78% of the time the prescribed procedures were followed in the robot-based intervention and 80.15% of the time the prescribed procedures were followed in the human-based intervention. The difference was significant, *t*(36) = 2.51, *p* < 0.02.

#### Post-test

The children in both conditions received the same post-test (i.e., ESCS) immediately (immediate post-test) and 1 month after intervention (delayed post-test). Different sets of toys were used in each of the post-tests.

#### Social validity

After the completion of the research, the parents of the children in both conditions were invited to complete the three-item social validity questionnaire. They used a 1-to-5 scale to rate: (1) the effectiveness of the robot-or human-based drama intervention in promoting their children’s IJA and RJA (1 as not effective at all and 5 as extremely effective); (2) the appropriateness of the program for their children (1 as not appropriate at all and 5 as extremely appropriate); and (3) their children’s ability to generalize the acquired social skills to their daily lives (1 as not able to generalize at all and 5 as extremely able to generalize). Each item was analyzed separately.

## Results

The children in both conditions completed all the drama sessions. We first looked at the number of times children were prompted when asked questions during training. They were prompted when they did not react during their turn for longer than 3 seconds. Our results showed that, on average, the children in the robot-based intervention were prompted 1.73 times (SD = 1.23) and those in the human-based intervention were prompted 2.81 times (SD = 1.98) across all sessions, *t*(36) = 2.34, *p* < 0.02. Overall, the proportion of time children responded accurately to the questions raised by the human researcher in the robot-based and human-based interventions was 0.75 (SD = 0.34) and 0.61 (SD = 0.27), respectively, across all sessions, *t*(36) = 2.12, *p* < 0.04.

We then compared the number of times children responded to joint attention (RJA) and initiated joint attention (IJA) before and after intervention in both conditions. Before doing so, we identified the possible covariate(s) that might influence the intervention outcomes. Therefore, we examined the correlations among children’s autism severity, intellectual functioning, RJA and IJA (see Table 2). Since we conducted ESCS before and after intervention, we averaged their ESCS scores across pre-tests and post-tests for this correlation analysis. Our findings showed that only autism severity measured by ADOS-2 was negatively correlated with both average IJA and average RJA. Therefore, we entered ADOS-2 as a covariate.

**Table 2:** Correlation among IJA and RJA scores and assessment performance of the participating children.

	Average RJA	Average IJA	SRS-2^nd^ Edition	SCQ	CARS™-2	ADOS-2	KBIT
Average RJA	--	0.698 ***	−0.30	−0.38	−0.27	−0.32*	−0.54
Average IJA	--	--	−0.31	−0.29	−0.12	−0.35*	−0.016

RJA: Response to joint attention measured in ESCS; IJA: Initiation of joint attention measured in ESCS; ***p < 0.001; *p < 0.05.

**Figure 3:**
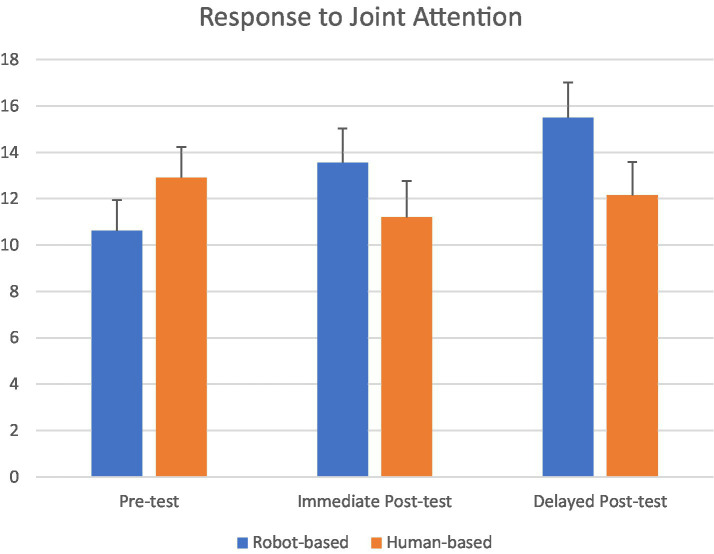
The number of RJA behaviors in children in robot-based intervention (*N* = 19; 4 females) and human-based intervention (*N* = 19; 3 females). Y-axis represents the number of times children responded to joint attention. The delayed post-test was administered 1 month after the intervention.

Figure 3 depicts the RJA results (the number of times children responded to joint attention) in the pre-test and two post-tests of the ESCS regarding the children with ASD in both conditions. Repeated measures ANOVA with Condition (Robot, Human) as the between-subject independent variable, Test (Pre-test, Immediate Post-test, Delayed Post-test) as the within-subject independent variable, the number of instances of RJA behavior as the continuous dependent variable, and ADOS-2 as the covariate, were conducted. The results showed a non-significant Test effect, *F*(2, 68) = 0.74, *p* < 0.48, *η*^2^ = 0.02, non-significant Condition effect, *F*(1, 34) = 0.48, *p* < 0.49, *η*^2^ = 0.01, significant covariate effect, *F*(1, 34) = 5.59, *p* < 0.024, *η*^2^ = 0.14, and significant Time × Condition interaction effect, *F*(2, 68) = 3.38, *p* < 0.04, *η*^2^ = 0.12. Bonferroni pair-wise comparison found a significant difference between the pre-test and delayed post-test in the robot-based intervention, *p* < 0.03, but not between the pre-test and immediate post-test, *p* < 0.09, nor between the immediate and delayed post-tests, *p* < 0.21. Different findings were reported in the human-based intervention. There was no significant difference between the pre-test and post-tests, *p* < 0.56. These findings suggested that robot-based intervention, but not human-based intervention could significantly enhance RJA. The enhancement, however, was evident in the delayed post-test rather than in the immediate post-test.

**Figure 4:**
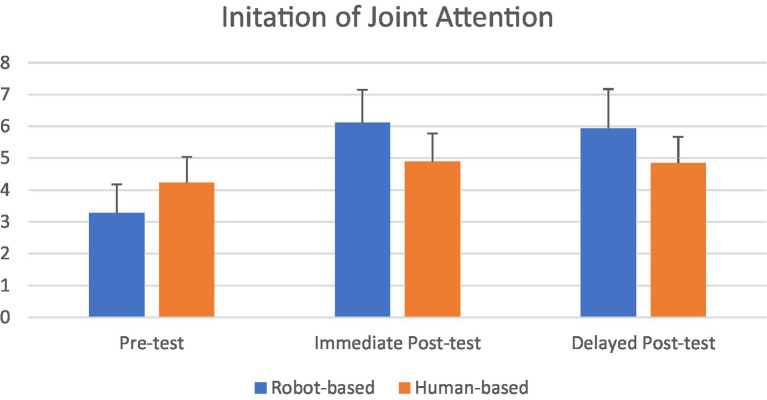
The number of IJA behaviors in children in robot-based intervention (*N* = 19; 4 females) and human-based intervention (*N* = 19; 3 females). Y-axis represents the number of times children initiated joint attention. The delayed post-test was administered 1 month after the intervention.

Figure 4 shows the IJA results (i.e., the number of times children initiated joint attention) in the pre-test and two post-tests of the ESCS. As for RJA, repeated measures ANOVA with Condition (Robot, Human) as the between-subject independent variable, Test (Pre-test, Immediate Post-test, Delayed Post-test) as the within-subject independent variable, the number of instances of IJA behavior as the continuous dependent variable, and ADOS-2 as the covariate, were conducted. The results showed a non-significant Test effect, *F*(2, 68) = 1.38, *p* < 0.26, *η*^2^ = 0.04, non-significant Condition effect, *F*(1, 34) = 0.001, *p* < 0.97, *η*^2^ = 0.00, significant covariate effect, *F*(1, 34) = 2.54, *p* < 0.12, *η*^2^ = 0.07, and significant Time x Condition interaction effect, *F*(2, 68) = 4.14, *p* < 0.02, *η*^2^ = 0.15. Bonferroni pair-wise comparison found a significant difference between the pre-test and immediate post-test, *p* < 0.03, and delayed post-test, *p* < 0.04 in the robot-based intervention. There was no significant difference between the two post-tests, *p* < 0.63. In contrast, in the human-based intervention, there was no significant difference between the pre-test and post-tests, *p* < 0.84. These findings suggested that robot-based intervention, but not human-based intervention, could significantly enhance IJA as well. The enhancement was evident in the immediate post-test and maintained in the delayed post-test.

Both groups of parents then completed the three-item social validity questionnaire. Using a Likert scale of 1 to 5, parents of the children in the robot-based intervention rated the effectiveness of the program in promoting IJA and RJA (*M* = 4.83, SD = 0.38), the appropriateness of the program (*M* = 4.83, SD = 0.38), and the program’s generalizability (*M* = 4.72, SD = 0.57). Those of the children in the human-based intervention rated the effectiveness of the program in promoting IJA and RJA (*M* = 4.00, SD = 0.95), the appropriateness of the program (*M* = 3.90, SD = 0.94), and the program’s generalizability (*M* = 3.95, SD = 0.92). The differences were significant: promotion of JA, *t*(36) = 3.49, *p* < 0.001, Cohen’s *d* = 1.12; appropriateness, *t*(36) = 3.91, *p* < 0.001, Cohen’s *d* = 1.25; and generalizability, *t*(36) = 3.07, *p* < 0.004, Cohen’s *d* = 0.99. These findings suggested that parents evaluated the robot-based intervention as more effective than the human-based intervention in promoting JA and helping their children to generalize the acquired skills to their daily lives.

## Discussion

While human-based applied behavior analysis (ABA) has been widely provided in many schools and treatment clinics, such intervention method relies heavily on human therapists and parents who are in charge of delivering interventions and assessing outcomes. Previous theories have proposed that ASD individuals may not be responsive to human therapists [Social Motivation Theory of Autism; (1)] and may have excessive reactivity and experience intensive sensory processing when interacting with humans [Intense World Theory; (2)]. In addition, human-based intervention incurs high costs to families with children with ASD. This has given rise to the application of technology, including social robots, in autism therapy. Yet, little is known about the effectiveness of robot-based intervention, in comparison to human-based intervention, in improving social and communication skills in children with autism. A few studies examined whether robot-based intervention is effective in promoting IJA and RJA and reported inconclusive findings (26, 28, 46). To date, very few studies have compared the effectiveness of robot-based intervention to that of human-based intervention, but they did not control the contents and procedures of both interventions (30, 31). This study aimed to examine whether the robot-based intervention was more effective than the human-based intervention in promoting RJA in autistic children with high support needs (IQs around 40), and if so, whether the robot-based intervention would also yield a positive impact on IJA. The findings of the present study contributed to the existing literature by showing that robot dramas were more effective in promoting IJA and RJA than human dramas, in which the contents and procedures of both kinds of dramas were the same.

During training, children in the robot-based intervention required fewer prompts than their peers in the human-based intervention. After taking six drama lessons, children in the robot-based intervention produced more RJA behaviors in the delayed post-test than in the pre-test. They were also found to have an increase in IJA behaviors in the immediate post-test compared to the pre-test despite IJA not having been explicitly taught. More promisingly, their increase was maintained in the delayed post-test. However, those improvements were not evident in the children taking human-based drama lessons. These findings were in line with parents’ ratings of both kinds of intervention. Parents of the children receiving the robot-based intervention rated the program more positively than those of the children receiving the human-based intervention, suggesting that the positive learning outcomes of the robot-based interventions could be generalized to human-to-human interactions.

Therefore, our findings have shown that the robot-based intervention was more effective than the human-based intervention in promoting RJA and IJA. These results are consistent with So et al.’s (28) study, which showed that the RJA of 18 autistic children with high support needs improved immediately after taking six robot drama lessons, and that these learning outcomes were maintained for at least 1 month. IJA was improved even though it was not explicitly taught. Our findings further support that IJA and RJA need not be taught separately or sequentially. Rather, ASD children could extract IJA skills from the dramas, where one of the social robots one did initiate joint attention in order to provide an opportunity for another robot to respond to it. More importantly, our work has extended the studies of So et al. (26, 28) by including a comparable human-based intervention, in which two human characters acted out the same dramas portrayed in the robot-based intervention. This comparison group was crucial for examining the effectiveness of robot-based intervention in improving JA. Different from the findings of So et al. (28), our findings have shown that parents of children receiving the robot-based (but not the human-based) intervention could promote their children’s JA and help them to generalize JA to other settings.

Nevertheless, the results of the present study contrast with the results of previous studies (30, 31, 46). Srinivasan et al. (30) and Srinivasan et al. (31) found that children in the human-based intervention and care-as-usual conditions performed better than those in the robotic condition. However, the components of the human-based intervention were more advanced and diverse than those of the robotic intervention, thereby making the two conditions incomparable. The components of both conditions should be tightly controlled such that their effectiveness can be compared. In the present study, we ensured that the intervention components (i.e., drama scripts) and prompting procedures were the same in both the human-and robot-based interventions. The only difference between the two conditions was the actors, which resulted in children’s different performance in RJA and IJA in the assessment.

Our findings are also different from those of Zheng et al. (46), who reported that robotic intervention did not enhance JA. Their participating children did not tend to respond to the robot’s joint attention bid, possibly because the robot did not engage with the children before and after the pointing and shifting of eye gaze. Rather, it merely commanded the children to shift their attention to one of the monitors (e.g., “Look over here!”). Therefore, the children might not have understood the robot’s intention in looking at and pointing in a particular direction. Our study resolved this issue by demonstrating RJA and its intention through robot dramas. In the first two dramas, one of our robots modeled eye contact with the other robot and demonstrated the importance of looking at each other (i.e., continuation of a conversation), because otherwise miscommunication can arise. This set the stage for teaching ASD children RJA behaviors in the last four dramas, in which the robot demonstrated the importance of following the eye gaze of the other robot so that both robots shared the common ground of the conversation. In each of these dramas, one of the social robots would correct the other robot if it failed to look at it or to shift its eye gaze to the target object. Through observing RJA behaviors modeled by the social robots, the children would understand the intention of an agent to look at a particular object during a conversation and know that RJA can have a positive impact on maintaining a conversation. Furthermore, merely watching the dramas might not be sufficient for children to acquire RJA. Therefore, during each drama, the human researcher asked the child to point to the objects the two robots were looking at. In order to respond accurately, the child needed to trace the robot’s eye gaze toward the target object, which helped the child to practice responding to others’ bids for JA. Teaching within a drama play may increase the likelihood of learning of JA skills and generalization of the acquired skills to the natural environment (47). Positive learning outcomes on symbolic behaviors and narration in autistic preschoolers with low support needs have also been reported in previous studies adopting a robot drama approach (26, 48).

One might contend that human actors can also model eye contact and demonstrate RJA in their dramas. Based on the empathizing-systemizing theory (6), human behaviors are rather complex and can be affected by various circumstances. In contrast, robots are operated on predictable and lawful systems regardless of circumstances. Since robots (but not humans) can be programmed in such a way that information is repeated in the same format, they are considered predictable and controllable, and thereby favorable for use with children with ASD. In the present study, the robots were programmed to perform the dramas such that each drama was presented consistently within a child’s lesson and across all the children’s lessons. It was crucial to deliver the dramas in the same way as designed in order not to confound the research. While our human actors were well trained before the intervention, it was challenging for them to perform the dramas in the same way each time. The findings from our fidelity checks also reported that the robots were more likely to follow prescribed procedures than the humans.

Nevertheless, in one of our previous studies on gestural intervention for autistic children with high support needs, we commented that human beings can serve as effective teaching agents if the lessons they deliver are highly structured (51). That study compared the learning outcomes in children with ASD and intellectual disabilities from robot-based intervention on gestural use to those from human-based intervention. Autistic children aged 6 to 12 with high support needs were randomly assigned to the robot group and the human group. During training, human actors or social robots demonstrated to the children 14 intransitive gestures using a highly structured and standardized intervention protocol (e.g., “*I would say bye-bye* (RIGHT HAND WAVES) *to the teacher after school*”). The findings showed that the children with ASD in the human group were as likely to recognize gestures and produce them accurately as those in the robot group. The learning outcomes of both groups were maintained for at least 2 weeks. Having said that, performing a drama consistently is more demanding (or less likely to be achieved) than simply demonstrating a gesture for human teachers.

Social robots have been widely used in therapy for individuals with ASD in the past decades (see reviews in 49, 50). According to a review by Huijnen et al. (51), robots are deployed in various domains in autism therapy including communication, social/interpersonal interactions and relations, play, emotional wellbeing, sensory experiences and coping, motor experiences and skills, preschool skills, and functioning in daily reality. Robots are found to arouse children’s interest and elicit their positive and productive responses, such as JA behaviors, self-initiated interactions, non-verbal communication skills, and ability to make eye contact (e.g., 52–54). Furthermore, they are more responsive and respond faster to feedback given by a technological object than that given by a human (e.g., 55). In their review, Begum et al. (56) even concluded that children with ASD are greatly interested in robots and interact with robots better than with humans.

While social robots may arouse autistic children’s interests and motivation and robotic intervention may facilitate their learning of social and behavioral skills, standardized intervention may not cater for the diverse learning needs of these children. Autism spectrum disorder is a complex neurodevelopmental disorder. Autistic individuals often have difficulties in social interactions and display restricted and repetitive behaviors (39), but the severity of those difficulties is heterogeneous. Such heterogeneity has been observed by scholars who were autistic themselves including Dr. Temple Grandin (who articulates the difference between autistic and non-autistic individuals as “Different, not less”) and Dr. Stephen Shore (who states, “If you have met one individual with autism, you have met one individual with autism”). A few studies have investigated heterogeneity of language skills in autistic children and identified homogeneous subgroups (e.g., 46, 57–59). In another study, Song and colleagues collected naturalistic language samples and identified four distinct subgroups among 50 Chinese-speaking autistic children aged between 4 and 8 years (58). Compared to other subgroups, Group 1, the least affected group, had the highest IQ, mild autism, and strongest verbal abilities. Group 2, the most severely affected group, had the lowest IQ, most severe autism, and weakest verbal abilities. Group 3 and Group 4 displayed moderate levels of verbal abilities and IQ. However, Group 4 had more severe autism than Group 3. Regarding language skills, Group 1 used fewer nouns and more pronouns than Group 2. In comparison to other groups, Group 1 also had the highest mean length of utterance (MLU). Likewise, based on the naturalistic language samples, another study also reported significant individual variations in MLU and word tokens in 42 Chinese-speaking autistic children aged 39 to 91 months (60). Heterogeneity is also observed in nonverbal impairments such as gesture production (61).

In the present study, standardized robotic intervention on joint attention was delivered. Given the heterogeneity of autism features, in future tailor-made robotic intervention should be designed and implemented. In order to accomplish this goal, social robots should be programed to be autonomous and social, meaning that they can communicate with the children as well as reacting to their behavior and conversation and adjusting the content of intervention. A study by de Graaf et al. (62) identified eight main social characteristics of a social robot and the capability of two-way interaction was one of them. For the domain of eye contact and joint attention, it is then crucial to develop non-invasive technologies for capturing gaze such as adaptation of computer vision systems (63) for more realistic deployment of the current system. Once the social robot detects the child is not looking at it, the robot can prompt and praise the child autonomously within established scenarios, alter behavior on the basis of parameters specified by the programmer, and process sensed data over time to understand the history of the interaction. Although autonomous social robots can be operated as the main teaching agents during the intervention, human therapists serve as important mediators in training. Human mediators can design and structure the learning environment in order to facilitate the generalization of acquired social skills to various scenarios. Additionally, human mediators can get involved in the training and assess whether the autistic children can immediately transfer the acquired social skills from the interactions with robots to human-to-human interactions.

While parents of the children in the robot-based intervention gave the program a positive rating, which suggests that the skills acquired in the robot-based intervention can be applied in daily life interactions with parents, there are still differences between the robot and human behaviors. For example, in addition to moving the eyes from one point to another, humans also produce subtle facial expressions when initiating or responding joint attention. Such kind of expressions were not demonstrated by the robots in the present study. Therefore, there are still technical constraints in using social robots to teach all sorts of social skills. This study has a few limitations. The first is our relatively small sample size for each condition, given that impairments in social skills can be heterogeneous (64). Also, the convenient sampling method may make the comparison with the generic ASD population not feasible. Second, our sample involved school-aged children with ASD. Since early intervention is recommended, future studies should examine whether robot-based intervention is still effective in improving IJA and RJA in preschoolers with ASD. Furthermore, it is critical to study whether the positive learning outcomes reported from the delayed post-test in the robot-based intervention can be maintained further. Although there are very few long-term follow-up studies (65), we may interview the parents regularly about changes in the JA skills of their children after completion of the research as well as observe the children’s interactions with parents and peers. Learning outcomes on joint attention can be examined by eye-tracker, which provide a reliable measure to support the qualitative assessment in ESCS. Moreover, fidelity score was lower in human-based intervention than in the robot-based intervention, resulting in different efficacy of the two interventions. Given the nature of human-based intervention, it was more challenging for the human teachers to follow the procedures tightly. Yet, the difference in the fidelity score between two conditions might be a confounding variable to the study. Finally, despite the technology advancement in developing and programming social robots, these robots and their maintenance may incur substantial costs to families of autistic children and schools. In order to reduce the financial burden of the users, one needs to lower the cost of robots, for example, by massive manufacture, while ensuring the durability.

To conclude, this study taught children with autism through robot-based or human-based dramas and found that children taking robot dramas could produce more joint attention behaviors than those taking human dramas. Parents also gave more positive ratings to robot dramas than human dramas. In future, researchers should examine whether robot-based intervention is more effective than human-based intervention in other aspects of social skills, such as conversation abilities, emotional understanding and expression, and narration. When researchers further develop robot-based interventions or empower those interventions by artificial intelligence, ethical and legal issues related to the data privacy, attachment of robots to children, and accountability of the decisions made by the robots may arise. Researchers should get human teachers involved as much as possible in designing and implementing robot-based interventions and inform the caregivers and educators of children with autism that robots are playing an assisting rather than a leading role in the classroom.

## Data availability statement

The raw data supporting the conclusions of this article will be made available by the authors, without undue reservation.

## Ethics statement

The studies involving human/animal participants were reviewed and approved by the Survey and Behavioural Research Ethics (SBRE) Board of the Chinese University of Hong Kong, in compliance with the Declaration of Helsinki (reference no. 14600817). We obtained the parents written informed consent prior to the start of the study. The participants provided written informed consent for the publication of any identifiable images.

## Author contributions

W-CS contributed to the conception and design of the study, performed the statistical analysis, wrote the first draft of the manuscript, and wrote sections of the manuscript. W-WL, and C-HC organized the database. CL programmed the robot. K-CN, F-YK, H-WL, and K-YL managed the data collection. All authors contributed to the article and approved the submitted version.

## Funding

This research has been fully supported by grants from the Regional Grant Council (Project no: GRF14606518). General Research Fund.

## Conflict of interest

The authors declare that the research was conducted in the absence of any commercial or financial relationships that could be construed as a potential conflict of interest.

## Publisher’s note

All claims expressed in this article are solely those of the authors and do not necessarily represent those of their affiliated organizations, or those of the publisher, the editors and the reviewers. Any product that may be evaluated in this article, or claim that may be made by its manufacturer, is not guaranteed or endorsed by the publisher.

## Footnotes

^1^Raw score for SRS was reported as there was no difference in SRS raw score between females and males for both groups.
